# A Case of Empyema Successfully Treated by an Operation

**Published:** 1845-05

**Authors:** W. C. Sneed

**Affiliations:** Frankfort, Kentucky


Art. IV.—*A Case of Empyema successfully treated by an Operation.* By W. C. Sneed, M.D., of Frankfort, Ky.

Empyema is defined by writers to be a collection of fluid in the pleural cavity, whether it be water, pus or blood. Pus being the most common, and that which is the most fatal in effects, is the one to which the term is most usually applied. This effusion may take place into the pleural cavity from various sources, and the lungs from their yielding structure readily give way to it. Vomica of the lungs, abscess of the mediastinum, or phlegmonous abscesses of the lungs and liver, may gradually, by perforating the tissues which separate them from the pleural cavity, discharge their contents into it, which they seem peculiarly disposed to do, from the tendency to a vacuum produced in the thorax during the act of inspiration. A subacute inflammation of the pleura partaking of a latent character, in which the sympathies are so obtuse as to give no evidence of its existence, or an acute inflammation which in its early or later stages ceases to excite pain and to disturb the action of the organs involved, instead of disappearing entirely, may degenerate into the chronic form, and thus give rise to a morbid secretion and deposit within the pleural cavity. The pleura partakes of the character of a mucous membrane, and when thus affected discharges, *gradatim*, a purulent fluid analogous in its character to that discharged in leucorrhœa, gonorrhœa, &c. In both the latter instances the secretion may be exhausting to the system, but is never directly fatal, in consequence of the secretion being carried off through the natural passages, and hence all the disadvantageous consequences are comprised in its formation. In the former they there only commence. The retention of the fluid is the danger to be dreaded, and constitutes the essence of the disease termed empyema.

In common pleurisy there is always a greater or less amount of serum secreted in the course of the disease, and deposited in the pleural cavity, which is clearly distinguishable by any one accustomed to the use of the stethescope, by the characteristic sound of egophony to which it gives rise. This secretion is usually removed by absorption during the recovery of the patient, leaving the thorax as free as before the attack. But in other cases, and where the disease is but illy cured, or the patient is too soon re-exposed to cold, a purulent secretion takes the place of the serous, and may accumulate in a large quantity in one of the cavities of the chest.

When collections of this character occur in one of the pleural cavities, the lung is compressed and retracts towards the spine, gradually yielding to the effused fluid that space which it formerly occupied. The vessels of the lung can no longer admit the fluids which filled them in a natural state. They become obliterated, adhere together, and the surface of the lung unites to the pleura costalis. The lung continues to diminish in size, and its internal adhesions multiply in proportion to the increase of the effused fluid. Finally, the substance of the lung disappears entirely; the bronchial pedicles become contracted, and the air cells totally obliterated. The lung grows incapable of being dilated, and if the effused fluid is evacuated the space which it occupied remains vacant. Nature being unable to fill up this vacuum by the expansion of the lung adopts other means to overcome the existing difficulty. When the disease has advanced to this stage we may expect but little from an operation. If, however, it is performed at an earlier period, and before the foregoing condition has supervened, it may be attended with success. Nature in some instances becomes her own surgeon, and forms an opening through the pleura pulmonalis into the bronchi of the lung, and the matter is discharged by expectoration. Relief obtained in this way may only be temporary, for the air in respiration finding its way into the cavity of the chest becomes a source of irritation, the oxygen acting upon the pus, and the patient in a short time sinking from exhaustion.

Another process of nature, often attended with success, is a disposition to point externally, and form an opening through the parietes of the chest. This is nearly as effectual as a surgical operation, with the exception that there is more danger of admitting the air, which taking the place of the fluid becomes a source of irritation in the manner before explained.

Sir A. Cooper says that he has never known a case to recover after the operation, and Dupuytren asserts that in more than fifty cases in which he had operated, not more than two had recovered. Later writers than the above have, however, given more favorable reports, and many cases are recorded by English surgeons in which the operation has been attended with success. (See *Cyclopædia Prac. Med., Art.* Empyema.)

In reporting the following case we introduce many details which must be tedious and uninteresting to those who have had an opportunity of witnessing similar cases, and have made themselves familiar with the treatment of this form of disease. But since no case of the kind has ever been reported in any Western journal, that we have any knowledge of, and as it was one of extreme interest to us, we feel disposed to give our notes of it just as we wrote them during its progress, believing that in this way we shall render it most instructive.

William Milam, aged 13 years, was attacked with pleuro-pneumonia on the 8th day of March, 1844. On Sunday, the 11th, we saw him and found that his attack was one of extreme violence, and required the prompt use of antiphlogistic remedies. He had been bled by his father, and had taken a dose of calomel on the day previous to our visit. We prescribed calomel in small doses combined with ipecacuan, and solution of tart. ant. in quantities to nauseate and keep down the febrile action. We saw him every day until the 18th, when the violence of the symptoms seemed to be subdued, and he gave every evidence of a speedy convalessence.

During the progress of his case we placed a large blister on the chest over the diseased lung, and kept it open by slightly irritating dressings, until we supposed his lung had sufficiently recovered to admit of its drying up. This seemed to have a powerful effect in checking the inflammation, and we hoped by keeping it open that it would, by its continued irritating effects, promote the speedy resolution of the lung and cause absorption of whatever effusion might have taken place in the pleural cavity. His case was aggravated and protracted by the existence of a large number of intestinal worms, which caused delirium, subsultus, &c., until they were expelled. By the proper administration of vermifuges, upwards of sixty ascaris lumbricoides were expelled, affording much relief and entire composure to the patient.

The case having progressed, and as we supposed, terminated, so favorably, we were much surprised on being summoned to visit him again on the first of April following. We found him laboring under the following symptoms, viz.: slight difficulty of breathing, with a hacking cough; pain in the left side; no expectoration, and considerable acceleration of pulse—about 120 in a minute.

The blistered surface it was found on examination had not healed up. Through and around the blistered surface there were a great number of small boils, very tender and exceedingly annoying to the patient. Ordered a starch poultice to the blistered surface, calomel and ipecacuan to subdue the excitement, to be followed by sulphate of morphine to lessen pain.

April 2. The difficulty of breathing still continues; pain in his left side when he coughs; respiration hurried; pulse 130 per minute, quick and small; lies on his left side altogether; no appetite; has 84 small boils on the left half of his body and limbs, some of them are the size of a small acorn, but the greater number are very small and painful, some have matured and burst, but the remainder are red and hard. They are more numerous in and around the blistered surface than elsewhere. We discovered no enlargement of the side, and did not suspect effusion of any description within the pleural cavity. Same prescription ordered.

April 5. Found him free from pain in the side, and much easier than when last visited, in consequence of the entire disappearance of the boils. They have left no other evidence of their existence except a small, hard, bluish looking spot. His side is evidently swollen, but the swelling seems to be confined to the skin and subcutaneous cellular tissue. There is considerable œdema under the blistered surface; the skin there red, and rather warmer than in other portions of the body; his general symptoms much improved; appetite good; bowels moved without medicine; skin moist; pulse not so frequent. Continue the anodyne.

7th. Since our last visit no material change has taken place in his general symptoms; the œdema has increased and now occupies a large portion of the left side; the skin is very red, and there are several hard spots about the centre of the swelling; the intercostal spaces are pointed at several places; side is evidently enlarged, and all the symptoms go clearly to prove the existence of a fluid in the pleural cavity; there is complete dullness over the whole of the left side; neither vesicular nor bronchial respiration can be heard from any portion of the lung. On measurement the side is found to be three inches larger than the opposite.

All the evidences of empyema being present, we had no hesitation in deciding upon the course proper to be pursued; but as we were not prepared to perform the operation, and the patient being six miles from town, we were obliged to postpone it until next day.

8th. Found the patient free from pain, cheerful, and in every way in a condition for the operation. The left side has increased in size since yesterday; breathing rapid but not difficult; the impulse of the heart is in the right side opposite its proper location; pulse 140; skin soft and pleasant; tongue clean; lies on his left side altogether; when turned upon his right side, becomes greatly distressed, and has to be turned back immediately.

In performing the operation we adoped the plan pursued by Dupuytren and Baron Larrey, which consists in making a valvular opening through the skin, by which means the air is prevented from entering the cavity of the chest after the fluid is evacuated.

We had the patient placed upon a trunnel-bed, that being the most convenient arrangement we could make, and punctured the side between the sixth and seventh ribs as near the angle, as the margin of the latissimus dorsi would permit. The skin being drawn upwards as tight as it could be, an incision about three-fourths of an inch long was made through it and the upper margin of the rib felt for. The lancet, which I used in preference to a trocar, was then gently passed into the cavity of the chest, and upon its withdrawal pus followed in great abundance. An assistant kept the orifice open by pressing the two edges apart, and the pus was suffered to flow out gradually as the expansion of the lung took place. The patient became exceedingly alarmed and we were compelled to close the orifice before we had taken more than half a pint of pus. The skin was then drawn down and the orifice completely closed with an adhesive strip without the introduction of a particle of air.

11th. The patient being composed and every thing favorable, we introduced a silver female catheter and drew off half a gallon of pure bland pus. This gave marked relief and he expressed himself greatly relieved from the difficulty of breathing and weight in his left side. His cough which had been more or less troublesome, ceased almost entirely, and he could turn on his right side without the sense of suffocation which he had before experienced so violently.

The heart could be felt under the sternum, and respiration could be distinctly heard in the upper portion of the lung. Not more than a few bubbles of air entered the cavity on the withdrawal of the catheter. In all the lower portion of the lung there was no sign of respiration, except a peculiar sound produced by the expansion and contraction of the organ, somewhat resembling the sound made by shaking a cask partly filled with water. This sound we attributed to the agitation of a portion of the pus left after the catheter was withdrawn.

The orifice was again closed by adhesive strips, and a bandage was applied round the chest and drawn as tight as he could well bear it. He was put to bed and ordered tincture opii sufficient to relieve pain and produce sleep. Strong hopes were entertained that the bandage would contribute much towards bringing the ribs down to their proper position, and that the lung when relieved of its pressure would expand and speedily reoccupy its former space. This expectation was to some extent realized; on our next visit we found that the ribs had descended to a considerable extent, and that the lung had expanded at its upper portion so as to be distinctly heard.

Pus, it may be proper to remark, when thus collected in the chest, unlike the serous effusions, is never or scarcely ever removed by absorption. The successive increase of the fluid reacts upon the membrane, which forms it, and thus keeps up a perpetual irritation and a perpetual flow. If not withdrawn from the body, it compresses the lungs against the mediastinum, and destroys the patient by suffocation, or wears him out by the irritation. In this case suffocation would have resulted in a few days, and the patient must have perished but for the timely removal of the fluid.

16th. Found patient cheerful and much improved since our last visit. His side has contracted and is but little larger than the opposite. His appetite is good and his food digests well; his bowels have been evacuated daily without the use of medicine; His skin is moist, but he has no cold stage or fever during the day. Respiration can be heard at the upper portion of the lung anteriorly, but not posteriorly. The incision has closed by adhesion and requires some force to pass the catheter into the chest.

Introduced the instrument and drew off three half pints of pure bland pus, without the introduction of any air. Ordered bandage to be continued and to be tightened as fast as the contraction of the chest took place. Continue tr. opii *pro re nata.*

29th. Visited patient every 2d and 3d day since last report and drew off about three half pints of pus each visit. His side continued to contract slowly, but the contraction has taken place posteriorly more than it has anteriorly. This we suppose has been in some measure owing to his resting on that portion of the ribs more than on the anterior. His appetite continues good, and his digestion undisturbed. He is greatly emaciated and when the pus is suffered to collect in larger quantities, he has chills and hectic fever. His heart is still in the right side and beats so forcibly as to be felt between the ribs. The right side has increased in fulness, and there is increased resonance in the right lung. This increased resonance has resulted from the extra amount of service required to be performed by this organ. The pus is becoming somewhat fœtid, and is filled with flakes of white matter which clog the instrument and render the extraction of the pus much more tedious and difficult. The ribs from the contraction of the side are in contact with each other, and the passage of the catheter between them is very troublesome.

In consequence of the sinking down of the ribs, the orifice has lost its valvular character and now passes directly into the cavity of the chest. We determined consequently to let it grow up and make another, after the plan pursued in the first instance by which the introduction of air, now unavoidable, would be prevented, and this was accordingly done and the difficulty obviated.

May 2d. Drew off nearly two quarts of pus, more fœtid but thinner than heretofore. General condition of the patient nearly the same as at our last visit.

6th. The matter has continued to form and is rather on the increase. It has become exceedingly fœtid and much thinner than it was at first. His father has introduced the instrument and drawn off every other day about three pints of fœtid pus. The rapid and continued secretion of pus has caused emaciation to progress very rapidly, and the patient is not able to walk across the house. His appetite continues good, and his food digests without difficuly. The fulness in front continues, and the instrument can be passed about three inches in the direction of the apex of the heart; pulse 140; skin hot and dry.

We have been giving him wine, tinct. mur. ferri, quinine, &c., but perceiving no good to result from their use they have been discontinued. He has been taking a geneious diet, which being well digested has supported the immense waste of the system and kept him alive.

9th. The pus has been accumulating since our last visit, and has distended the side and pressed against the diaphragm, until the abdomen is considerably enlarged; introduced the instrument and drew off two quarts of thin pus; after evacuating the matter he expressed himself relieved from the pressure on the stomach, of which he had been complaining for several hours. His breathing was much improved, and his feelings in every way greatly alleviated.

We discovered several days since a collection of air under the pectoralis major of the left side, which has continued to increase until it is now occupying a space as large as the palm of my hand. When pressed upon it disappears, but returns immediately on removing the pressure. When the pus is withdrawn from the thorax the emphysema disappears, and does not return until the cavity is again filled with pus. There is a communication from the thorax to the under portion of the pectoral muscles, which has been produced by an ulceration through the intercostal muscles.

We have used a great variety of medicines with the view of keeping up the tone of the system, and to arrest, if possible, the immense secretion of pus. So far as we have been able to judge, nothing has been of material service to him except the tinct. opii, which has relieved pain and produced sleep.

14th. His father has introduced the catheter night and morning and drawn off each time on an average about half a pint of pus. He has been allowed animal food freely. His strength has increased under the use of it, and in every way he seems much improved. The character of the pus continues about the same.

The instrument has to be passed about two and a half inches upwards and inwards before the pus will flow out. There is evidently a false membrane formed within the pleura costalis. The emphysema still exists when the pus is suffered to accumulate, but disappears when it is drawn off.

We continued to visit our patient once or twice a week during the months of May and June, and pursued about the same course of treatment as that above detailed. We discontinued the use of all kinds of medicine and relied entirely upon wine and a generous diet to support the system. The matter was drawn off daily, and often twice a day, by his father, averaging from one to three half pints per diem. The bandage was kept on and gradually tightened as the side contracted. Under the use of the wine and a generous diet he gained strength notwithstanding the copious secrection of pus, and by the first of July he could walk across the house without help.

July 1st. He is much improved in every way. The pus is not so fœtid nor secreted in such abundance; it is more transparent, and has now more the appearance of serum than of pus. The emphysema has nearly disappeared, and the side has contracted until it is about two inches smaller than the opposite one.

The absence of all inflammatory symptoms and the change in the character of the secretion induced us to believe, that much might be effected by the use of astringent injections into the pleural cavity, and we determined, notwithstanding the doubts expressed by the highest authors upon the subject, to try their efficacy in the case of our patient. Before resorting to this remedy we learned that a medical gentleman in an adjoining county had been the subject of the same affection in his youth, and we addressed him a letter inquiring as to the treatment pursued in his case. In reply to our inquiries he addressed us a letter, which is subjoined, giving a brief history of his case, and recommending very strongly the use of astringent injections.

We commenced injecting about half a pint of a weak decoction of oak bark into the cavity once a day, after having first drawn off the pus. The result was truly gratifying. From this time he improved rapidly; the secretion lost its fœtor, and became almost serous in a few days. It continued to be secreted, however, until about the middle of August, when the amount became so small and serous that we discontinued the injections, and let the orifice close. The emphysema gradually subsided after we commenced the use of the injections, and entirely disappeared before the orifice closed. His left side is at this time two and a half inches smaller than the right. The spine is curved to the right about three-fourths of an inch. His left shoulder instead of sinking, as I anticipated it would, is higher than the right: this may be attributed to the fact of his lying on his left side altogether duing his illness. The heart is now under the sternum and can be felt about as distinctly in the left as in the right side. Respiration can be distinctly heard through a greater portion of the left lung, and the metallic tinkling which has existed so long has ceased entirely.

The gratifying results of the treatment of this case of empyema—results so seldom met with when paracentesis is performed, appear to be due, in part, to the youth of the subject, and to the great recuperative powers of his system. But much we think is due also to the valvular opening which was made, and which kept open for so long a period, enabled us to prevent the immense secretion from accumulating in the chest, in consequence of which the lungs were permitted to expand and the ribs to contract until the cavity had become obliterated. In the numberless introductions of the catheter, a bubble or two of air was the most ever allowed to enter the pleural cavity, and these at the withdrawal of the instrument. The temperature of the atmosphere seemed to exert a very decided effect upon the secretion; whenever there was a change in the weather, the air becoming cold, his symptoms were invariably aggravated; the pus became more copious, and the fœtor was increased.

This case, altogether, we consider a very remarkable one, for its violence, and for the speediness of the cure. From the commencement of his illness to the time of the first operation, only about thirty days elapsed, and from the day of the operation to the closing of the orifice, about four months transpired. During this time the instrument was introduced upwards of one hundred times, and we feel fully assured that not less than fifteen gallons of pus and serum were taken from the chest of the patient little sufferer.

We saw our patient to day, and find him in the enjoyment of good health. The heart is in its proper place, and respiration can be heard through the whole of the left lung. The side is still contracted, but no inconvenience is felt in consequence of the diminution of the lung. The cure may be considered perfect.

Frankfort, April 1, 1845.

The following is the letter referred to in the preceding article:

OWENTON, Ky., July 10th, 1844.

*Dr. Sneed:*

DEAR SIR—In reply to your letter inquiring for the particulars of my case, I hasten to reply, that I was tāken with dysentery when about 13 or 14 years of age. The physicians gave me medicine which checked the disease suddenly and threw me into violent pain, which settled in my left side. I knew nothing for three days; from this time, or shortly afterwards, there was some swelling perceived in my left side, which gradually increased for sometime, when I was assured that matter had formed in the chest, but I refused to submit to an operation until I could see more appearance of it externally.

The pulsation of my heart was found in the right side, and Dr. Moore, of Shelbyville, and others, supposed that malformation of the chest existed, and that the heart had never occupied its proper situation. My side was finally opened, the incision into the chest being about one inch below the left nipple, and the opening through the skin about an inch below that. There was half a gallon of pus discharged before the opening was dressed, and I think as much more that night. During the time the pulsation of the heart moved several inches towards the left side.

In the course of about twelve months after this, a small tumor made its appearance two inches below the incision. This was also opened and discharged freely. About this time, or perhaps sooner, I began to expectorate a very offensive pus, often, in the morning, as soon as I began to move in bed, discharging a pint of so acrid a character that it seemed to excoriate the throat. Sometime after this, the communication became divided into the cavity of the chest. Every time that I breathed, the air would pass in and out at the opening with such violence that it could be distinctly heard, and its effects seen upon the discharge. When I coughed this sound could be heard across the room.

My physician now resorted to an injection, consisting of a decoction of barks—a part of which I usually coughed up, while a portion was discharged at the lower opening in my side, proving that the cavity of the sack was common to the two outlets, and that there was adhesion of the lungs to the side, and a direct communication between them and the external openings. After using the injection a few times I coughed up no more matter; the lower orifice in my side healed, and I gradually recovered. A probe for a long time could be passed five and a half inches directly towards the spine. I do not know that any medicine was of much advantage to me. My appetite was good all the time, which being indulged, supported the system and kept me from sinking. For a time dieting was attempted, but I grew worse every day, when the system was abandoned, and I was allowed bacon, beef-steak, and such nourishing articles. My side ran for two years, and I have no doubt that the discharge of matter from the two openings, and by coughing, amounted to sixty gallons. This statement may seem unreasonable, but I believe that my estimate is rather under than over the mark. Very little air now passes through the lobe of my left lung.

Yours, most respectfully,

F. REED.

